# Propofol Exposure in Pregnant Rats Induces Neurotoxicity and Persistent Learning Deficit in the Offspring

**DOI:** 10.3390/brainsci4020356

**Published:** 2014-05-06

**Authors:** Ming Xiong, Jing Li, Hussain M. Alhashem, Vasanti Tilak, Anuradha Patel, Sergey Pisklakov, Allan Siegel, Jiang Hong Ye, Alex Bekker

**Affiliations:** 1 Department of Anesthesiology, Rutgers-New Jersey Medical School, 185 South Orange Avenue, Newark, NJ 07107, USA; E-Mails: jingli_umdnj@yahoo.com (J.L.); hashem.h.m@gmail.com (H.M.A.); tilakva@njms.rutgers.edu (V.T.); patelan@njms.rutgers.edu (A.P.); pisklase@njms.rutgers.edu (S.P.); ye@njms.rutgers.edu (J.H.Y.); bekkeray@njms.rutgers.edu (A.B.); 2 Department of Psychiatry, Rutgers-New Jersey Medical School, 185 South Orange Avenue, Newark, NJ 07107, USA; E-Mail: siegel@njms.rutgers.edu

**Keywords:** propofol, prenatal exposure, neurotoxicity, learning deficit, offspring

## Abstract

Propofol is a general anesthetic widely used in surgical procedures, including those in pregnant women. Preclinical studies suggest that propofol may cause neuronal injury to the offspring of primates if it is administered during pregnancy. However, it is unknown whether those neuronal changes would lead to long-term behavioral deficits in the offspring. In this study, propofol (0.4 mg/kg/min, IV, 2 h), saline, or intralipid solution was administered to pregnant rats on gestational day 18. We detected increased levels of cleaved caspase-3 in fetal brain at 6 h after propofol exposure. The neuronal density of the hippocampus of offspring was reduced significantly on postnatal day 10 (P10) and P28. Synaptophysin levels were also significantly reduced on P28. Furthermore, exploratory and learning behaviors of offspring rats (started at P28) were assessed in open-field trial and eight-arm radial maze. The offspring from propofol-treated dams showed significantly less exploratory activity in the open-field test and less spatial learning in the eight-arm radial maze. Thus, this study suggested that propofol exposure during pregnancy in rat increased cleaved caspsase-3 levels in fetal brain, deletion of neurons, reduced synaptophysin levels in the hippocampal region, and persistent learning deficits in the offspring.

## 1. Introduction

Although general anesthetics have been used safely for surgery in adults for decades [1], their use in pregnancy is becoming a more concerning issue due to their potential effects on the immature or developing brain [2,3,4]. Non-obstetric surgeries occur in 0.15% to 2% of pregnant women, involving approximately 75,000 in the US and 76,000 in Europe [5]. Anesthetic administration during pregnancy would lead to a significant amount of general anesthetic exposure to the fetus and its long-term effects on human fetal brain development remain unclear. More laboratory animal studies, especially *in vivo* system, are needed in this field to verify the link between general anesthetics early exposure to immature brain and the possible neurotoxicity.

The effects of the inhalational general anesthetic on fetal brain development have been extensively studied in animal models by multiple laboratories. Pregnant rats administered isoflurane on gestational day 14 (G14) had birthed pups that subsequently showed significant deficits in spatial working memory as adults [6,7]. Development of spatial learning occurs predominantly in the hippocampus [8,9] and involves synaptogenesis—the process of forming connections between neurons. Synaptogenesis correlates with the expression of several types of proteins including the synaptic vesicle proteins (synaptophysin). Synaptophysin is an essential element of the exocytosis of synaptic vesicles [10,11], which is treated as a marker of synaptic density [12]. Conversely, neuronal nuclei antigen (NeuN) is exclusively expressed in mature, post-mitotic neurons with mature synapses [13,14]. Impaired learning of the pups treated on G14 was associated with lower synaptophysin expression in the hippocampus [7]. Furthermore, a more recent study suggests that anesthetics transiently and differentially affect dendritic spines and filopodia [15]. In contrast, isoflurane administered on a later gestational day, G20, in a pregnant rat model did not impair learning or memory in the adult offspring [16]. Thus, the effect of prenatal exposure of isoflurane on spatial learning is still controversial and may depend on the gestational age.

Propofol (2,6-diisopropylphenol) is the most common intravenous (IV) general anesthetic agent in current clinical practice. Recent evidence suggests that propofol, like isoflurane, may cause neurotoxicity to young developing brains [17,18,19,20]. Creeley *et al.* [20] recently demonstrated that propofol general anesthesia administered to pregnant rhesus monkey induced apoptosis in the brain of fetal monkey. However, that study focused on the acute histological changes of the fetus brain caused by administration of general anesthesia to dams rather than the effect of sub-anesthetic (sedative) dose of propofol. It has been reported that propofol even at a sub-anesthetic dose caused widespread neuroapoptosis in the neonate and led to a persistent decrease in the dendritic growth in cultured GABA neurons [17,21]. Therefore, we hypothesized that the use of sub-anesthetic dose of propofol during pregnancy may interfere with fetal brain development and lead to abnormal behavior and learning disabilities in the offspring. To test this hypothesis, pregnant rats were sedated with propofol (low activity but intact righting reflex) on G18. Since it is unknown whether a sub-anesthetic dose of propofol (sedation but not surgical plane) administered to pregnant rats can induce apoptosis in the brains of fetal rats, caspase-3 activation was assessed in the brain of fetal rats 6 h after propofol infusion. We followed the pups prenatally exposed to propofol to postnatal day 28 and compared the offspring brain at P10 and P28 for the neuron density, maturity of neurons (NeuN), and synaptophysin levels in CA1 and CA3 regions of the hippocampus to those of offspring from saline-exposed pregnant rats. To investigate the long-term effect of prenatal propofol exposure on rat brain development, the ability for learning and memory in offspring (P28) were assessed with the open field and the eight-arm radial maze, which previously have detected correlations between hippocampal alterations and spatial learning deficits [22,23].

## 2. Materials and Methods

### 2.1. Animals

All experiments were approved by the IACUC of Rutgers-New Jersey Medical School. Pregnant Sprague-Dawley (SD) rats (Taconic Farms, Germantown, NY, USA) and their offspring were housed in standard polypropylene cages in a temperature and humidity regulated room. They were exposed to a 12 h light-dark cycle with access to standard rat chow and water ad libido. Pregnant SD rats were acclimated to the approved housing facility for 3 days before anesthetic treatment on gestational day 18 (G18). To minimize confusion, *N* refers to number of dams or litters and *n* refers to number of offspring (pups).

### 2.2. Propofol Administration

Pregnant rats were randomly assigned to receive IV infusion of propofol, 2 mL of intralipid or 2 mL of normal saline infusion for 2 h via tail vein on day 18 of pregnancy. Propofol was obtained from APP Pharmaceuticals, LLC (Schaumburg, IL, USA). Intralipid 20% IV fat emulsion was obtained from Baxter (Baxter Healthcare Corporation, Deerfield, IL, USA). On gestational day 18 (G18), the pregnant rats were gently restrained to facilitate insertion of a 24 gauge IV catheter in the lateral tail veins. After confirming the catheter was in the vein, the catheter was secured in place with HY-Tape (Allegro Medical Supplies Inc., Bolingbrook, IL, USA) and attached to a T-connector extension set (Baxter Healthcare Corporation, Deerfield, IL, USA) for the continuous target control infusion of propofol, intralipid, or normal saline. The rate of propofol infusion was adjusted to 0.4 mg/kg/min so it induced sedation but not a general anesthesia (low activity but intact righting reflex). The final dose of propofol was approximately 18–20 mg.

To decrease the effects of stress caused by monitoring the vital signs and metabolic state of dams on fetus, a separate group of dams (*N* = 6) was inserted with two single 24-gauge IV catheters, one into each lateral tail vein. One catheter was used for the continuous target control infusion of propofol, and the second was used for drawing venous blood at 0, 15, 30, 45 and 60 min during propofol infusion. The blood was analyzed for pH, PvCO_2_, PvO_2_, HCO_3_^−^ and blood glucose levels (iStat analyzer, Abaxis, Union City, CA, USA). The arterial oxygen saturation, pulse strength, heart rate, and breath rate were continuously monitored during propofol infusion, by using the Pulse Oximeter (Harvard Apparatus, Holliston, MA, USA). Maternal blood pressure was monitored by non-invasive blood pressure system (Harvard Apparatus, Holliston, MA, USA). In all studies of the propofol group, the rat rectal temperature was monitored and maintained at 37 ± 0.5 °C with heating lamp and temperature controller (Harvard Apparatus, Holliston, MA, USA).

The control dams received an infusion of normal saline or intralipid for the same duration. It was not feasible to apply available monitors to the control group. However, we closely observed the activity of the dams to ensure that the pregnant rats were tolerant of the infusion of intralipid or normal saline. In order to avoid stress to pregnant dams and maintain the IV infusion, the controls were placed in infusion cages (Harvard Apparatus, Holliston, MA, USA) that allowed the rats to move freely. We applied soft rodent cervical collars onto the control rats to prevent the rats from biting and removing the IV during the 2 h infusion. The control group rats were tolerant of the 2 h infusion with minimum abnormal behavior. The pups from the group used for monitoring vital signs and blood gas were excluded from the following histological and behavioral studies.

After infusion, we randomly divided pregnant rats into two groups. Group 1: Fetuses were removed via cesarean section (C/S) at 6 h after propofol, saline or intralipid infusion for study of apoptosis. Group 2: pregnant rats were allowed to undergo normal spontaneous vaginal delivery (NSVD) (usually 48–72 h after propofol or saline infusion). In the NSVD groups (6 pregnant rats from propofol infusion, 6 pregnant rats from normal saline), pregnant rats were returned to their respective cages and were allowed to completely recover from infusion and deliver pups normally. There was no significant effect of maternal propofol administration on average litter size (total number of neonates: control 12.3 ± 0.8, propofol: 12.3 ± 0.8). The neonates did not show any sedative behavior after birth. Therefore, there were sufficient numbers of offspring pups for the histology (*n* = 6 per arm) and behavioral studies (*n* = 12 to 18 pups per group).

### 2.3. Western Blot Analysis for Cleaved Casepase-3 in the Fetal Brain

Previous study has indicated that propofol general anesthesia to pregnant rhesus monkey induced apoptosis in the fetal brain [20]. However, it is unknown whether a sub-anesthetic dose of propofol (sedation but not surgical plane) administered to pregnant rats can induce apoptosis in the brains of fetal rats. Western blot analysis was used to measure the levels of activated caspase-3 in fetal brain tissues from pregnant rats treated with saline (*n* = 3), intralipid (*n* = 3) or propofol (*n* = 3). At 6 h after saline, intralipid or propofol infusion, C-section was performed under chloral hydrate (400 mg/kg, IP) anesthesia in group 1. The fetuses were immediately euthanized with chloral hydrate, and were perfused intracardially with 0.9% saline. The fetal brains were collected and stored at −80 °C for Western blot analysis. Brains of two fetuses from each pregnant rat were studied.

Western blot analysis was performed as described [7]. Half cerebral hemispheres were used for Western blot analysis. Protein concentration was determined by the BCA method with bovine serum albumin as the standard. Briefly, protein samples (50 μg) were separated by 12% sodium dodecyl sulfate polyacrylamide gel electrophoresis (SDS-PAGE) and transferred to a nitrocellulose membrane. The membranes were blocked by nonfat dry milk buffer for 1.5 h and then incubated overnight at 4 °C with primary antibody against cleaved caspase-3 (17–19 kDa) resulting from cleavage at aspartate position 175 (1:1000; Cell Signaling Technology, Danvers, MA, USA). The membranes were incubated with horseradish peroxidase-conjugated secondary antibodies and developed with ECL kit. Equal protein loading in each lane was confirmed by GADPH levels, detected by hybridization with a 1:2000 dilution of anti-GAPDH antibody (1:2000; Sigma-Aldrich, St. Louis, MO, USA). The results were expressed as a relative density to GAPDH. The optical densities of bands were quantitatively analyzed by using ImageJ version 1.38 (NIH, Bethesda, MD, USA). We quantified the Western blots in two steps. First, we used GAPDH levels to normalize protein levels (e.g., determining the ratio of cleaved to GAPDH amount) and to control for loading differences in the total protein amount. Second, we presented changes in protein levels in rats undergoing propofol or intralipid as a percentage of those in the saline control group.

### 2.4. Neonatal Brain Histology Studies

To test whether prenatal propofol exposure affected the numbers and maturation of neurons after birth, Nissl staining and neuronal nuclei antigen (NeuN) immunohistochemistry analysis were used to assess neuronal density in the hippocampus. Control (*n* = 6 from pregnant rats treated with saline) and propofol (*n* = 6 from pregnant rats treated with propofol) offspring rats were randomly selected and euthanized with chloral hydrate on P10 and P28 respectively, and were perfused intracardially with 0.9% saline followed by the fixative (4% paraformaldehyde in 0.1 M phosphate buffer, pH 7.4). The brain was extracted from the skull, postfixed (overnight, 4 °C) and cryoprotected (24 h, 4 °C, 30% sucrose in 0.1 M phosphate buffer, pH 7.4). Coronal sections (30 µm) from hippocampal region were cut on a freezing microtome (Microm HM 550, Walldorf, German). Sections were collected for Nissl staining and immunohistochemistry study.

### 2.5. Nissl Staining

Nissl staining was performed as previously described [24]. After Nissl staining, the neuronal cells of the hippocampal CA1 and CA3 pyramidal cell layer were identified by using an assisted image analysis system, consisting of an Nikon Eclipse 80i bright field microscope (Micron Optics, Cedar Knoll, NJ, USA) interfaced with a color digital camera Nikon DS-Ri1 digital camera (Micron Optics, Cedar Knoll, NJ, USA), and a computer with a NIS-Elements BR 3.0 software (Micron Optics, Cedar Knoll, NJ, USA). Cells were counted under a high magnification (400×). Every fifth dorsal hippocampal section between −3.8 and −4.3 Bregma was counted (five sections per rat; 6 rats per group). In each section, neuronal cells were counted in 1 mm-length of hippocampal CA1 and CA3 areas. Only intact neurons with a clearly defined cell body and nucleus were counted. Bilateral values were obtained by pathologists blinded to the treatment, averaged, and reported as absolute number of cells per area counted.

### 2.6. Immunohistochemistry Analysis for NeuN

The methods for immunohistochemistry of mature neurons were performed as described in our previous studies [25,26]. Nonspecific staining in the brain sections were minimized by sequential treatment with 3% hydrogen peroxide solution and 0.1 M PBS containing 0.5% Triton X-100, 3% normal goat serum and 3% bovine albumin (1 hr). Tissue sections were incubated overnight at 4 °C with the primary monoclonal mouse anti-NeuN antibody (EMD Millipore, Temecula, CA, USA), diluted 1:500 in 0.1 M PBS containing 1% normal goat serum, 1% bovine albumin and 0.1% Triton X-100. Subsequent reagents included biotinylated goat anti-mouse IgG secondary antibody (Vector, Burlingame, CA, USA), Vectastain Elite ABC reagents (Vector), and diaminobenzidine in sodium acetate. The stained sections were rinsed, mounted on gelatin-subbed slides, dehydrated, and protected with cover slips. Quantitative measurement was performed as the method in Nissl staining.

### 2.7. Immunofluorescence Staining of Synaptophysin in the Hippocampus

Previous study indicated that sevoflurane administered to pregnant mice (G14) reduced synaptophysin levels in the hippocampus in fetal and offspring mice [27]. To test whether propofol administered to pregnant rats affected the levels of synaptophysin in the offspring, immunofluorescence staining was used to assess synaptophysin levels in the hippocampus in P28 pups. Brain tissues collected from P28 rats (control: *n* = 6; propofol: *n* = 6) was processed as aforementioned. Brain sections were blocked with blocking solution for 1 h at room temperature and incubated overnight at 4 °C with anti-synaptophysin (1:500 in blocking solution, Sigma-Aldrich, St. Louis, MO, USA). After several rinses, sections were incubated with a fluorescent-conjugated anti-rabbit secondary antibody (1:200; Sigma-Aldrich, St. Louis, MO) for 1 h in the dark at room temperature. Wet mounted sections were viewed immediately. Semiquantitative analysis for the density of synaptophysin staining was conducted on the photographs by using ImageJ version 1.38 (NIH, Bethesda, MD, USA), as described in the previous study [27].

### 2.8. Animal Behavioral Studies

Given that propofol administered to pregnant rats can induce caspase-3 activation in the fetal brain, neuronal deletion, and reduced synaptophysin levels in the hippocampus in the offspring up to the age of postnatal 28 days, propofol exposure *in utero* may affect the memory and learning ability of the juvenile offspring rats. To test this hypothesis, the open-field task and eight-arm radial maze were used to assess the memory and learning ability at P28 rats according to the previous studies [22,23].

### 2.9. Open-Field Test

A large Plexiglas box (120 cm × 80 cm × 40 cm high) had outlines of 30 squares (large grid, 6 × 5 equal blocks) on the bottom to track the movement of each rat during each trial. The 18 squares on the perimeter and the 12 squares in the center area were called the outer squares and inner squares, respectively. The control (*n* = 18 from pregnant rats treated with saline) and propofol exposed (*n* = 16 from pregnant rats treated with propofol) offspring rats on P28 were allowed to run an open-field test under a red light. The investigator and assistant remained still at approximately 0.5 m away from the apparatus during all trials. Each trial was recorded with a mounted video camera and investigators blinded to the group scored each trial for the number of entries made into each outer square and each inner square; percentage of time spent in the inner squares; the number of wall rears, and number of fecal boli.

### 2.10. Eight-Arm Radial Maze

The standard eight-arm radial maze (Med Associates Inc., St. Albsan, VT, USA) was used in an isolated dark room between 13:00 and 15:00 h to assess rat learning and memory of offspring on P28. A dustless precision pellet (Bio-Serv, Frechtown, NJ, USA) was used as a reinforcer. All rats were food restricted to maintain 85% of their baseline weight; rats gained about 5 g (body weight) per week. As described in Liu and Bilkey [28], control (*n* = 12 from pregnant rats treated with saline) and propofol (*n* = 16 from pregnant rats treated with propofol) offspring rats received seven sessions of habituation (one session per day) on postnatal days 28 to 34, before training for the radial 8-arm maze. During habituation, reinforcers were scattered randomly along the arms and central platform. Rats were placed onto the central platform and allowed to adapt to the maze for 2 min each day, then the doors were automatically opened, and rats were allowed to explore the arms for 10 min. The session ended when 10 min had elapsed. On the last two days of habituation (day 6, 7), one reinforcer was placed at the end of each arm. After habituation, all rats were trained on the standard radial arm maze task for 5 consecutive days (one session per day). Each training session had a single reinforcer at the end of each arm. Each training session ran until (a) all eight arms had been chosen; (b) 5 min had elapsed since the start of the test; or (c) 2 min had elapsed since the rat’s last choice [23,28]. The following data in each session were recorded: (a) the number of errors (entering a previously visited arm); (b) the number of correct choices prior to first error; and (c) the total time to complete entering all eight arms. The maze was wiped clean with 75% alcohol between rats.

### 2.11. Statistical Analysis

Data from eight-arm radial maze were subjected to a two-way repeated measures analysis of variance (RM ANOVA), and was followed by Tukey’s *post hoc* analysis when a significant overall main effect was found (*p* < 0.05). Student “*t*” test or one-way ANOVA was used in open-field test and staining results. Physiologic parameters were analyzed using a repeated measure one-way ANOVA. Statistical significance was declared at *p < 0.05*.

## 3. Results

### 3.1. Physiologic Parameters of Dams during Propofol Infusion

**Figure 1:**
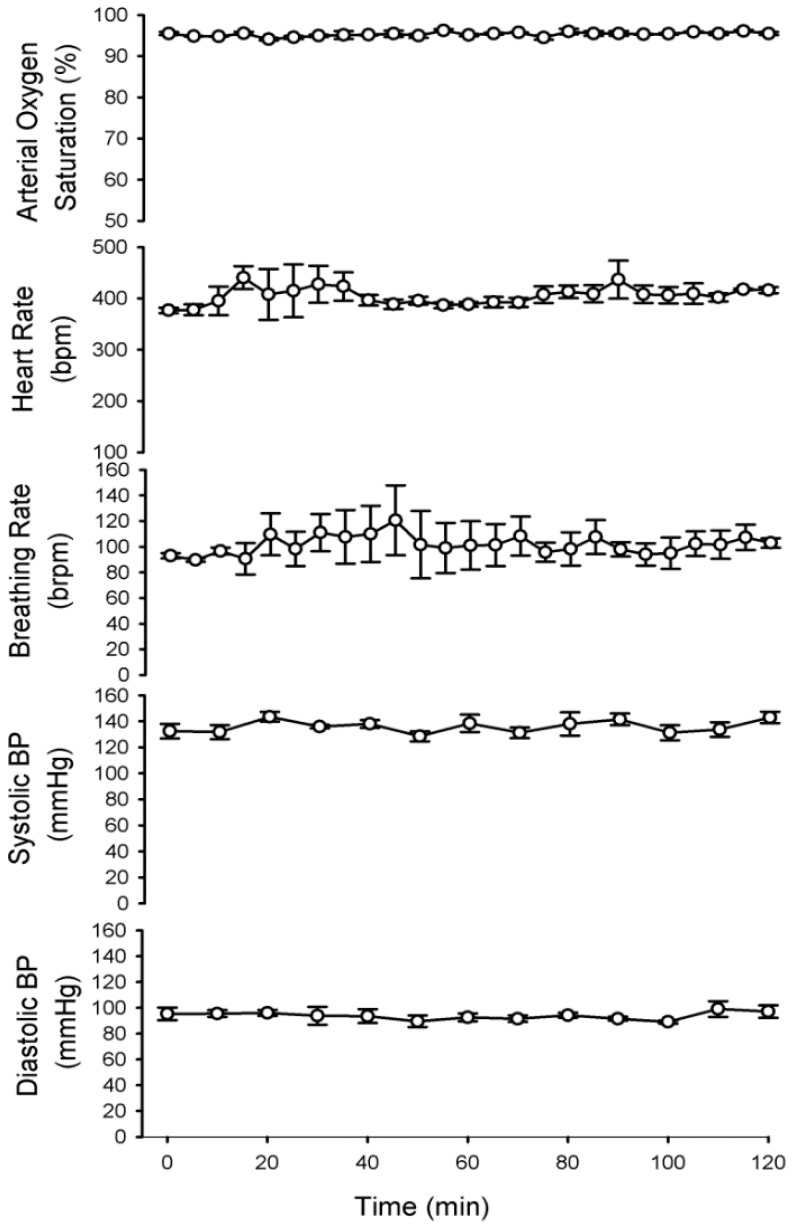
Propofol infusion did not cause significant disturbances in the vital signs of pregnant rats. Arterial oxygen saturation, heart rate and breathing rate were monitored by pulse oximeter. The data were averaged every 5 min during propofol exposure. Blood pressure was measured every 10 min by non-invasive blood pressure system. These data indicated no significant differences in any of the measured parameters compared to the baseline (0 h) (*N* = 6).

In the propofol infusion group, the maternal vital signs (arterial oxygen saturation, pulse distention, heart rate, and breath rate) were continuously monitored and averaged every 5 min. We did not detect any significant change of physiological parameters at any point during the 2 h infusion (Figure 1). The maternal blood pressure was measured every 10 min. Systolic blood pressure and diastolic blood pressure did not differ throughout the 2 h infusion. There were no signs of metabolic or respiratory distress. PvCO2, PvO2, HCO_3_^−^, and pH did not differ significantly from baseline (awake rats) (Table 1). Taking these measures and maintaining the vital signs reduced the possibility that propofol-induced neurodegeneration in the fetal brains was caused by physiologic side effects (e.g., hypoxia, hypo- or hyperglycemia and hypertension).

**Table 1:** Maternal venous blood gas and glucose levels during propofol infusion.

VBG	Propofol infusion		
	0 h (awake)	1 h	2 h
pH (venous)	7.40 ± 0.00	7.38 ± 0.03	7.33 ± 0.00
P_V_CO_2_ (mmHg)	40.1 ± 4.5	42.5 ± 2.5	41.6 ± 2.4
P_V_O_2_ (mmHg)	50.3 ± 7.3	50.3 ± 2.8	73.0 ± 14.1
HCO_3_^−^ (mmol/L)	22.7 ± 3.2	26.7 ± 1.5	24.6 ± 2.4
Na^+^ (mmol/L)	146.0 ± 4.0	136.6 ± 2.0	140.6 ± 2.3
K^+^ (mmol/L)	3.7 ± 0.2	3.6 ± 0.2	3.7 ± 0.3
Glucose (mg/dL)	89 ± 1.2	83.4 ± 1.2	87.0 ± 5.5

Data are presented as mean ± SEM. Propofol infusion rate at the range of 0.4 mg/kg/min did not affect venous blood gas values and blood glucose levels significantly. *N* = 6 dams. PvCO2 = venous carbon dioxide tension; PvO2 = venous oxygen tension, VBG = venous blood gas.

### 3.2. Cleaved Caspase-3 Levels in the Brain of Fetal Rats

**Figure 2:**
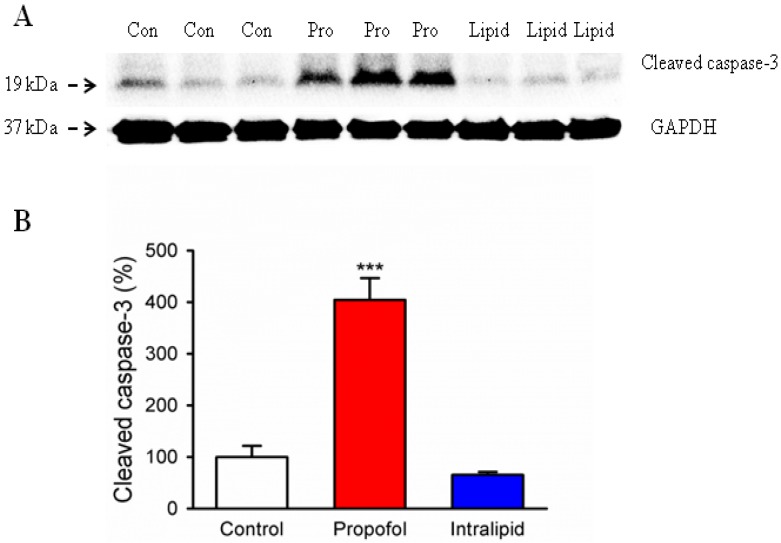
Prenatal propofol exposure induced caspase-3 activation in the brain tissues of fetal rats. A 2 h IV infusion of Propofol (Pro), intralipid (Lipid) or saline control (Con) was administered to pregnant rats on G18 (*N* = 3 dams per group). Fetuses were removed via C-section at six hours post infusion. Whole cerebral hemispheres of fetal rats were harvested and analyzed by Western blot. (**A**) Propofol infusion induced significantly higher caspase-3 activation in the brain tissues of fetal rats as compared to the control condition or intralipid infusion with Western blot analysis. There is no statistically significant difference in the amounts of GAPDH in the rat brain tissues following the propofol, intralipid treatment or control condition. (**B**) Quantification of the Western blot shows that propofol anesthesia increased cleaved caspase-3 levels in the rat brain tissues compared to the control groups (*** *p* < 0.001). Intralipid infusion did not affect the levels of cleaved caspase-3 in the brain of fetal rats. Data from six pups (2 pups from 3 litters) per group are expressed as means ± SEM.

Immunoblotting showed that propofol treatment of the dams induced denser visible bands of cleaved caspase-3 in the brains of the fetuses than the control conditions (Figure 2A). The GAPDH levels were similar among these three groups. Quantification of the Western blot showed that the propofol treatment significantly increased cleaved caspase-3 levels in the brain tissues of fetal rats compared to the control condition (404% ± 42.23% *vs.* 100% ± 21.58%, *p* < 0.001) (Figure 2B). Intralipid treatment slightly, but not significantly, decreased cleaved caspase-3 levels in the brain tissues of fetal rats as compared with that in the control group (65% ± 5.59% *vs.* 100% ± 21.58%; Figure 2B). Considered together, these results suggested that propofol treatment for 2 h in pregnant rats, but not the propofol intralipid carrier, induced caspase-3 activation in fetal rat brains.

### 3.3. Neuron Deletion in the Hippocampus of Offspring Rats

**Figure 3:**
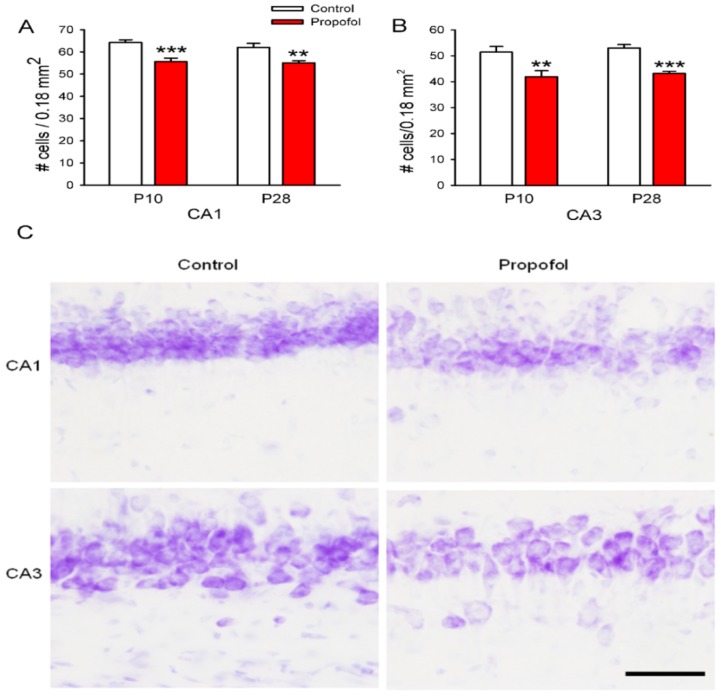
Propofol administered to pregnant rats induced a significant loss of hippocampal neurons in the offspring rats. Quantification of the neuronal density counts in the hippocampal CA1 (**A**) and CA3 (**B**) regions of pups on postnatal day 10 (P10), and P28. Neuronal density counts were obtained from Nissl stained sections. Data from six pups per group (*N* = 6; 1 pup per litter, *n* = 6) are expressed as means ± SEM. ** *p* < 0.01, *** *p* < 0.001 significant difference from age-matched control group. (**C**) Representative photomicrographs of hippocampal sections stained with Nissl indicated neuron density in P28 offspring. Scale bar = 50 µm.

Next, we tested whether the propofol-induced neuroapoptosis in the fetal brain affected the numbers and maturation of neurons after birth. The neuronal density and matured neurons of the hippocampus of offspring on postnatal day10 (P10) and 28 (P28) were assessed by Nissl staining. The number of neurons in the hippocampus (CA1, CA3) of pups prenatally exposed to propofol for 2 h was significantly fewer than those in the saline treated group on P10 and P28 (Figure 3). The P10 pups prenatally exposed to propofol showed a 19% reduction in neuron number in CA3 and a 14% reduction in CA1 compared to the saline treated pups (Figure 3). Furthermore, the number of neurons in the CA1 and CA3 fields of the hippocampus of the exposed pups on postnatal day 28 was still significantly fewer than those of age-matched control pups (Figure 3, *p* < 0.01; *p* < 0.001). Our results indicated that prenatal exposure to propofol reduced neuron populations in the CA1 and CA3 regions of the hippocampus area. The neuron density had not recovered in these regions by P28.

### 3.4. NeuN Staining in the Hippocampus

**Figure 4:**
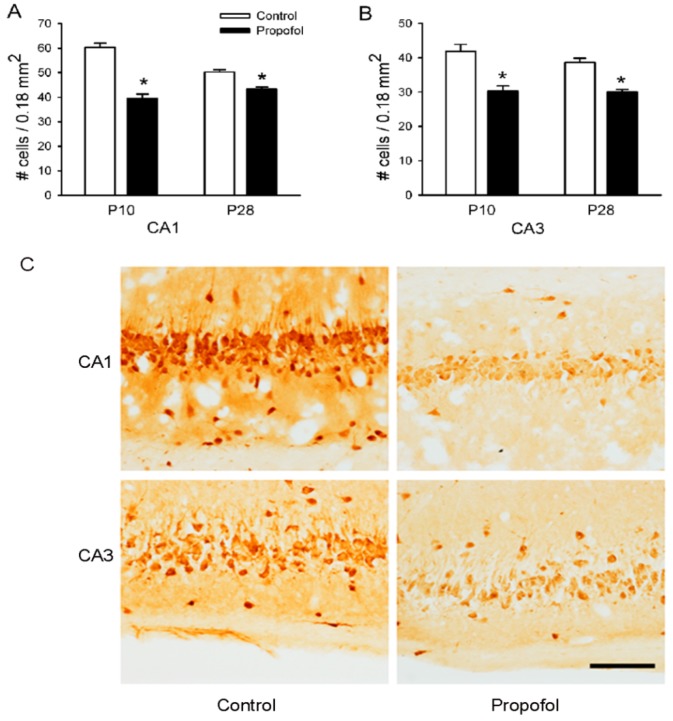
Propofol administered to pregnant rats reduced the number of NeuN-positive neurons in the CA3 and CA1 regions of the hippocampus of the offspring rats. The number of NeuN-positive neurons in the hippocampal CA1 (**A**) and CA3 (**B**) regions of pups on P10 and P28 were counted. Data from six pups per group (*N* = 6, 1 offspring per litter, *n* = 6) are expressed as means ± SEM. * *p* < 0.001 significant difference from age-matched control group. (**C**) Representative photomicrographs of NeuN-positive neurons in the CA1 and CA3 regions of pups on P28. Scale bar = 50 µm

Mature neurons are easily distinguished from glial cells by the presence of NeuN antigen [13,29]. To discern whether propofol affected the number of mature neurons, the NeuN expression was examined in the hippocampal sections of P10 and P28 pups by immunohistochemistry. The number of mature neurons (NeuN positive, dark brown particles) in the CA1 and CA3 regions of the hippocampus in the control group were significantly greater than those of the propofol prenatally exposed group on P10 and P28 (Figure 4. *p* < 0.001).

### 3.5. Synaptophysin Levels in the Hippocampus of Offspring Rats

**Figure 5:**
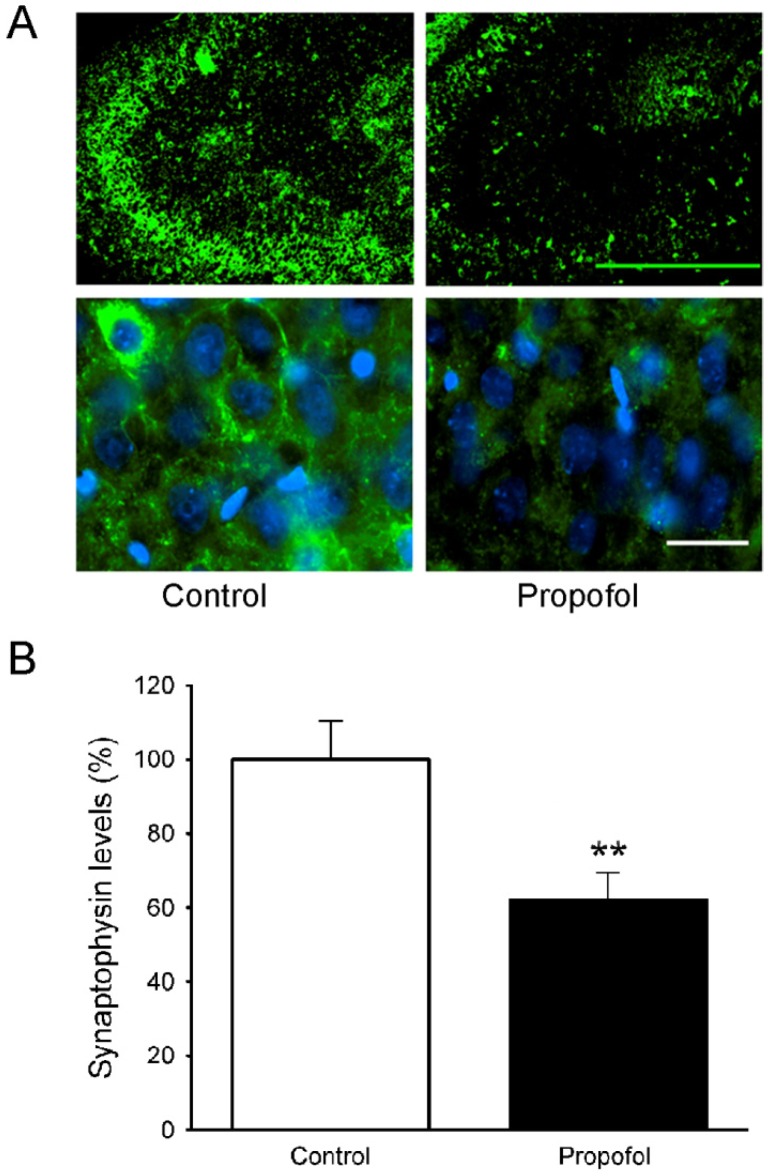
Propofol administered to pregnant rats decreased synaptophysin levels in the hippocampus of the offspring rats. (**A**) Immunofluorescent staining for synaptophysin in the brain tissues of offspring rats on P28. Left panel: control; right panel: propofol. Scale bar = 100 µm. (**B**) Semiquantitative analysis of the immunohistochemistry image shows that the synaptophysin levels in the brain of rats exposed to propofol *in utero* are significantly lower than that of control rats (** *p* = 0.009 significant difference from age-matched control group; *N* = 6 litters, 1 offspring per litter, *n* = 6 per group).

Synaptophysin is expressed by neurons and indicates their proliferation and extent of synaptogenesis. The effects of propofol administered to pregnant rats on synaptophysin levels in the brains of the juvenile offspring (P28) were assessed by immunofluorescence staining. The brightness of synaptophysin staining is very apparent in both low and high magnified lens (Figure 5A, left panel) in juvenile control rats (P28). However, juvenile rats (P28) from propofol-exposed group showed lower synaptophysin levels in the hippocampus (Figure 5A, right panel) than those from the saline treated dams. Semiquantitative analysis by Image J software indicated that the intensity of synaptophysin was significantly lower in the cells of the hippocampus of juvenile rats from the propofol-exposed dams (Figure 5B, control: 100.0 ± 10.4 *vs.* propofol: 62.1 ± 7.5, *p* = 0.009). Those results provided direct evidence that synaptogenesis, which should be very active at this age of rats, was significantly reduced in the juvenile rats (P28) exposed to propofol *in utero*.

### 3.6. Open-Field Test

**Figure 6:**
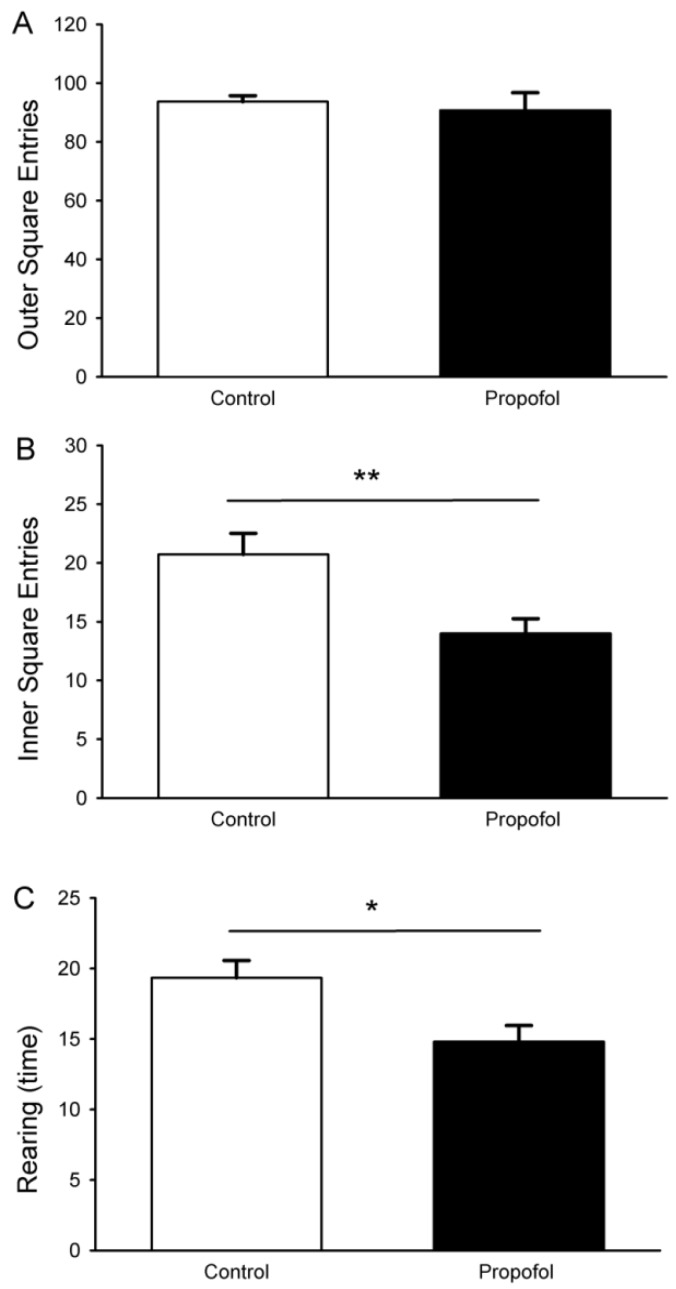
Effect of propofol administered to pregnant rats on the performance of offspring rats (P28) in the open field task. Pups exposed to propofol *in utero* made significantly fewer entries into the inner squares (**B**) and number of wall rears than age-matched control pups (**C**). No major difference in the outer square entries (**A**). Data are means ± SEM. Student *t* test. *N* = 6, 3 pups per litter, *n* = 18 for control; *N* = 6, 2–3 pups per litter, *n* = 16 for propofol group. For all figures, *asterisks* represent statistical difference (* *p* < 0.05; ** *p* < 0.01).

The open field test is a behavioral test that can assess the ability of small animals to explore a novel environment [22], and is able to detect significant differences associated with hippocampal-dependent behaviors. Pups that were prenatally exposed to propofol made a similar number of entries to the outer squares as control rats (Figure 6A). In contrast, the prenatally propofol exposed pups (P28) had significantly fewer entries into the inner squares (Figure 6B: propofol 76.7 ± 5.0, control 95.0 ± 4.1, *p* < 0.01) and fewer number of wall rears (Figure 6C: propofol 15.8 ± 1.3, control 20.1 ± 1.3; *p* < 0.05) in the open-field task than pups from control dams had. The time spent in the inner or outer squares and the number of fecal boli were similar between the two groups (Table 2). Thus, the propofol-exposed group showed less novel exploration in a new environment.

**Table 2:** Effect of prenatal propofol exposure on number of boli and time spent in the outer and inner squares in the open field task.

Open field test	Control (*n* = 18)	Propofol (*n* = 16)
Number of feces	4.0 ± 0.4	3.5 ± 0.4
Time spent in the inner squares (seconds)	29.0 ± 3.0	26.4 ± 3.3
Time spent in the outer squares (seconds)	271 ± 3.0	273.6 ± 3.3

Data are expressed as means ± SEM.

### 3.7. Eight-Arm Radial Maze

The radial eight-arm maze task is used to measure specific aspects of spatial working and reference memory in rodents [23,28]. It assesses the ability of animals to develop an optimal strategy for exploring their environment and obtaining food with minimal effort. Juvenile rats from both the control and propofol-exposed groups were randomly selected for the radial eight-arm maze task.

**Figure 7:**
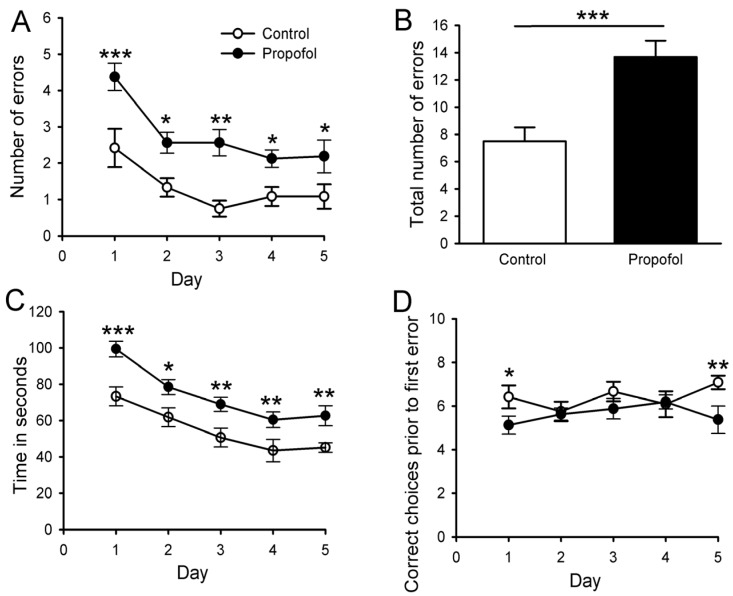
Propofol administered to pregnant rats induced impaired learning and memory ability in the offspring rats in the eight arm radial maze task. Rats exposed to propofol *in utero* made more errors relative to the controls across 5 days of testing (**A**). There was also a significant difference between groups in terms of the total number of errors over the 5 days of testing (**B**). Rats exposed to propofol *in utero* also took longer time to complete the maze (**C**) and made fewer correct choices prior to first error (**D**) than age-matched controls. Data are expressed as mean ± SEM. *N* = 6, 2 pups per litter, *n* = 12 for control; *N* = 6, 2–3 pups per litter, *n* = 16 for propofol group. * *p* < 0.05; ** *p* < 0.01; *** *p* < 0.01 *vs.* control. Two-way repeated measures analysis of variance followed by Tukey’s *post hoc* analysis.

Total number of errors: Juvenile rats prenatally exposed to IV propofol for 2 h made significantly more errors across 5 days of test than the saline controls (Figure 7A). A two-way repeated measure ANOVA revealed significant effects of group [*F* (1,104) = 28.89, *p* < 0.001], and day [*F* (4,104) = 8.19, *p* < 0.001]. The sum total number of errors throughout the 5 days of test in the prenatal propofol-treated group was significantly greater than those of the control group, [*t* (17) = 4.69, *p* < 0.001] (Figure 7B).

Total time to complete entering all eight arms: A two-way repeated measure ANOVA revealed significant effects of group [*F* (1,104) = 27.19, *p* < 0.001], and day [*F* (4,104) = 23.78, *p* < 0.001]. *Post hoc* analysis indicated that the juvenile rats prenatally exposed to propofol took significantly longer to enter all eight arms on days 1, 2, 3, 4 and 5 than the saline controls (Figure 7C).

Correct choices prior to the first error (Figure 7D): This variable indicates the number of correct choices prior to the first error across 5 days of tests in both groups. A two-way repeated measure ANOVA revealed significant effects of group [*F* (1,104) = 6.22, *p* = 0. 02], but no effects of the day (*p* = 0.59). The Tukey’s *post hoc* analysis revealed that prenatal propofol-treated pups made significantly fewer correct choices prior to the first error than the saline control pups on Day 1 and Day 5.

The group × day interaction was not significant for any of these variables.

In summary, our results revealed that the prenatal propofol exposure induced significant impairment in learning and spatial memory in juvenile rats as determined by the radial eight-arm maze task. The significant differences in two major indicators (number of errors and total time to complete the maze) detected deficits in the spatial working and reference memory of juvenile rats that were prenatally exposed to propofol.

## 4. Discussion

In the present study, we intravenously infused propofol into pregnant (G18) rats to study the neurotoxic effects of propofol in the offspring brain. We found that this manipulation caused: (A) a significant increase in the levels of cleaved caspase-3 in fetal brain 6 h after propofol exposure. (B) A significant reduction of the neuron population in the CA1 and CA3 regions of the hippocampus at P10 and at P28. (C) A substantial reduction in synaptophysin expression in the hippocampus in P28 rats. (D) Impairment of learning and memory in juvenile offspring prenatally exposed to propofol. These results suggest that prenatal propofol exposure may trigger apoptosis and neuron deletion in offspring rat, and may affect brain development and learning abilities of the offspring. In the current study, we focused on the effects of propofol exposure on the hippocampus since our initial Nissl staining indicated more obvious neuron deletion in the brains of the rat offspring. Hippocampus is well known as an important area for memory and learning development [8,9]. Using behavioral tests that had previously detected hippocampal alterations [22,23], we observed significant learning and memory deficits in the juvenile rats (P28) from the propofol-exposed dams in the open field test and the eight-arm radial maze task.

Several animal studies show that propofol exposure to young brain induces apoptosis [17,18,20,27]. The mechanism or causality of propofol induced neurotoxicity in immature brain remains unknown despite several studies that have revealed a possible molecular pathway [19,30,31]. The propofol exposure in rodent model is focused on neonate [17,18]. There is no information available about prenatal propofol exposure in rodent animal model. Our results are in agreement with the recent Creeley *et al.* [20] study in which significant numbers of apoptotic neurons were detected in fetal monkey brain after prenatal exposure of propofol. However, the current study revealed the effect of propofol exposure on rodent fetal brain, and also long-term effect of baby brain damage. Rodent animal models allow examination of the animal brains at several time points (such as 6 h post infusion (E18/E19), 10 days, 28 days after birth). We were able to conduct effective behavioral studies at P28 for long-term learning and memory deficits because of the larger litters of rodents (average 12–15 pups). Another unique feature of this study is that the method of administering propofol (0.4 mg/kg/min IV for 2 h slow infusion) induced an anesthetic state close to sedation (no movement but intact righting reflex) in the pregnant rats rather than a general anesthetic state (no intact righting reflex). There is some evidence that sub-anesthetic propofol exposure on baby mice triggers neuronal damage, but the damage was never shown in the fetal brain of the rodent animal [18]. Whether this dosage of propofol (0.4 mg/kg/min for 2 h) in rodents is relevant to clinical practice is very difficult to predict. Since clinical anesthesia with propofol can last for 6 h, future studies will focus on the effects of duration of infusion such as 30 min *vs.* 6 h and different rates of infusion to determine whether the total amount and/or the duration of propofol exposure alters the outcome of fetal brain damage in this rodent animal model.

Propofol has a high lipid solubility and can pass the placental barrier efficiently and be detected in fetal blood in a matter of a few minutes [32]. Propofol exposure to the fetal brain via the placenta raises several other potential issues. During the 2 h propofol infusion, the pregnant rat may develop hypotension, hypoxia, hypoglycemia, hypothermia, and/or acidosis which all may lead to placenta hypoperfusion, and possible fetal brain damage. These confounding factors were not detected by our closely vital sign monitoring and venous blood gas determinations, indicating those derangements may not be the cause of fetal brain damage.

It is a greater technical challenge to place IV catheter in small animals. However, it is important to study the intravenous medication (propofol) via intravenous routes because pharmacokinetics and pharmacodynamics of other routes of administration may differ. Stress induced by the procedures of this experiment such as placing a tail IV in the awake rats (pain from IV), and infusion propofol for two hours (stress from the environment) could be a concern. We did not detect any neuronal and behavioral change in our saline control group or the propofol carrier (20% intralipid), suggesting our results are likely due to propofol itself rather than the stress of an IV placement, or the amount of fluid infusion. We did apply a soft rodent cervical collar to prevent the pregnant rats from biting off the tail IV catheters and to allow the animals to run freely in a cage during 2 h infusion.

The activation of caspase-3, deletion of neurons and decreased synaptophysin in the hippocampus may be the factors contributing for learning and memory deficits in 28 day old offspring. In support, propofol (IP, 40 mg/kg or 20 mg/kg for 6 h) decreased synaptic spine density in 5-day-old rodents [33]. These synaptic spine density changes persisted into adulthood [33]. A recent study indicated that sevoflurane administered to pregnant mice (G14) also induced neuronal damage, disrupted synaptophysin in the hippocampus, and correlated to behavioral changes of the juveniles [27].

However, there are several limitations in our current studies. (1) Only one region (hippocamus) was evaluated in Nissls staining and immunohistochemistry studies. Our caspase-3 studies indicated the damage may not limited to one brain area. We selected hippocampal area as initial target of evaluation is the fact that hippocampal neuron deletion is correlated to memories function impairment in rodent behavioral studies [22,34]. In our future study, we would fully examine other brain areas; (2) Synaptogenesis in hippocampus is assessed with a semi-quantitative measure (Image J Analysis), the level of synaptophysin more or less may be associated with neuronal deletion in the same region. A more quantitative method (such as western blot) needs to be performed to assess the relationship between the prenatal propofol exposure and the synaptophysin decrease in the hippocampus region of offspring; (3) Additional behavioral tests are warranted to verify the overall long-term damage to fetal brain after a 2 h prenatal propofol exposure due to the chosen behavior tests complexity and different interpretations of those rodent behavioral tests.

A major health concern of an anesthetic exposure *in utero* is the potential long-lasting consequences. Our results are consistent with a number of studies that suggest the exposure to any one of the general anesthetics—isoflurane [6,7,16], sevoflurane [27], and ketamine [35]—to pregnant mammals has caused cellular or functional changes to the hippocampus in the neonates that affected behavior in the juveniles or adults. However, Li *et al.* [16] observed that isoflurane prenatally administered on G21 did not cause spatial learning deficits in the adult offspring. Thus, it remains controversial that embryonic brain development at G18 in rat is equivalent to G21. In addition, a different experimental approach and different general anesthetics may explain the difference. Our goal for current studies is to test intravenous propofol infusion model in small animal for prenatal propofol exposure in order to examine fetal brain damage (short and long term) in offspring rats. Additional well-designed experiments *in vivo* are required for detecting a causal link between prenatal propofol exposure and long-term behavioral change, and to elucidate possible ways to mitigate those changes in rats. Environmental enrichment of the offspring has reduced the long term neurotoxicity of neonatal sevoflurane exposure in mice [27]. We caution that animal studies cannot directly predict the toxicities in humans [36,37].

## 5. Conclusions

In conclusion, a propofol IV infusion to pregnant dams on G18 induces significant caspase-3 activation and neuronal deletion of hippocampal region. Our behavioral studies suggest that the memory may be abnormal at P28 in the offspring rats exposed to propofol on G18. Taken together, these results suggest that prenatal exposure to propofol at 0.4 mg/kg/min for 2 h at age of G18 may cause neural damage and behavioral deficits to the offspring rats.
